# Controlled Suspensions Enable Rapid Determinations of Intrinsic Dissolution Rate and Apparent Solubility of Poorly Water-Soluble Compounds

**DOI:** 10.1007/s11095-017-2188-1

**Published:** 2017-06-15

**Authors:** Sara B. E. Andersson, Caroline Alvebratt, Christel A. S. Bergström

**Affiliations:** Department of Pharmacy, Uppsala University, P.O. Box 580, SE-751 23 Uppsala, Sweden

**Keywords:** apparent solubility, controlled suspensions, dissolution-limited drug absorption, intrinsic dissolution rate, particle size reduction

## Abstract

**Purpose:**

To develop a small-scale set-up to rapidly and accurately determine the intrinsic dissolution rate (IDR) and apparent solubility of poorly water-soluble compounds.

**Methods:**

The IDR and apparent solubility (S_app_) were measured in fasted state simulated intestinal fluid (FaSSIF) for six model compounds using wet-milled controlled suspensions (1.0% (*w*/w) PVP and 0.2% (*w*/w) SDS) and the μDISS Profiler. Particle size distribution was measured using a Zetasizer and the total surface area was calculated making use of the density of the compound. Powder and disc dissolution were performed and compared to the IDR of the controlled suspensions.

**Results:**

The IDR values obtained from the controlled suspensions were in excellent agreement with IDR from disc measurements. The method used low amount of compound (μg-scale) and the experiments were completed within a few minutes. The IDR values ranged from 0.2–70.6 μg/min/cm^2^ and the IDR/S_app_ ratio ranged from 0.015 to 0.23. This ratio was used to indicate particle size sensitivity on intestinal concentrations reached for poorly water-soluble compounds.

**Conclusions:**

The established method is a new, desirable tool that provides the means for rapid and highly accurate measurements of the IDR and apparent solubility in biorelevant dissolution media. The IDR/S_app_ is proposed as a measure of particle size sensitivity when significant solubilization may occur.

## Introduction

In the development of new oral drugs, poorly water-soluble compounds remain a challenge for the pharmaceutical industry even though substantial efforts have been made to tackle this problem. The Biopharmaceutics Classification System (BCS) is widely used to categorize drug compounds into different classes (1). Previous investigations of marketed compounds estimate approximately 30% to be BCS class 2 compounds showing poor solubility but high permeability (2,3). However, the trend towards selection of lipophilic compounds during the drug optimization process has increased the proportion of BCS class 2 compounds in the drug discovery pipeline from ~30% to ~50–60% (4), but numbers as high as 90% have also been reported (3). Physicochemical properties of BCS class 2 compounds allow them to quickly permeate biomembranes, and for these compounds the dissolution rate and/or the solubility become the limiting factors to drug absorption. Since the transit time through the main absorptive site in the intestine is short, a low dissolution rate often results in low bioavailability of the compound. Therefore, methods to estimate drug dissolution and formulation strategies to improve drug dissolution are of interest.

The relationship between solubility, dissolution rate (dC/dt) and the surface area of a compound is given by the Noyes & Whitney equation (5):

1 $$ \frac{\mathrm{dC}}{\mathrm{dt}}={\frac{\mathrm{D}}{\mathrm{h}}}^{\ast }{\mathrm{A}}^{\ast}\left({\mathrm{C}}_{\mathrm{S}}-{\mathrm{C}}_{\mathrm{t}}\right) $$

where D is the diffusion coefficient, h is the thickness of the diffusion layer, A is the surface area, C_s_ is the saturated concentration and C_t_ is the concentration of the dissolved compound in the bulk at time t. Thus, for any given dose of a drug, a proportional increase in dissolution rate will occur if the total surface area of the solid drug is increased by reduction of the particle size. Particle size reduction is, therefore, one of the first approaches to explore to increase the dissolution rate and thereby the fraction absorbed.

Dissolution testing is used to assess the dissolution properties of the drug itself and to select suitable excipients of the formulation. It is also used as a tool to select the dosage form with the most appropriate and reproducible release profile (6). The dissolution rate is typically reported as the concentration (μg/mL) or as the percentage of the added solid material dissolved per time unit. A more standardized measurement of dissolution is the intrinsic dissolution rate (IDR), i.e. the surface specific dissolution rate (μg/min/cm^2^) in which the dissolution rate is adjusted for the surface area of the solid material in contact with water. This allows formulation strategies to be identified as it will clearly provide information on the effects that can be expected from e.g. particle size reduction, increased porosity or increased dispersion (deaggregation) in water. The IDR is also commonly measured during salt exploration and selection. Detailed description of the IDR can be found in chapter 1087 of the U.S. Pharmacopeia. The IDR-value should be reported together with the experimental conditions used, since solvent, temperature, laboratory equipment and experimental settings will impact the final measured value.

The IDR is typically measured from rotating discs of compacted powder in dissolution media where miniaturized alternatives can be used nowadays instead of the traditional USP-type apparatus (7,8). The feasibility of using disc IDR (DIDR) to determine the BCS class has also been investigated in a previous study, where a DIDR of 100 μg/min/cm^2^ was suggested to be a cut-off between soluble and poorly soluble drugs (9). An advantage with the miniaturized disc method is that the amount of solid material needed can be as little as 5 mg to make compacted discs, while traditional apparatus need a larger amount, up to 700 mg (7). This is a clear advantage as it renders the IDR measurements applicable in the early stages of drug development when the material available is limited. However, this method is usually time-consuming for BCS class 2 and 4 compounds where several hours (or even days) are needed to measure the IDR (10). This can be compared to BCS class 1 and 3 compounds where less than one hour is usually sufficient to determine IDR (7). The same trend was seen in a recently published paper where an IDR guide was established (10). In that paper, the authors concluded that, when the apparent solubility (S_app_) is greater than 1 mg/mL, the disc dissolution method is suitable and allows the IDR value to be determined within an hour.

Powder dissolution assays can be performed to speed up the dissolution process. In these experiments, solid powder, with a much larger surface area than a compacted disc, is applied straight into the vials, whereupon the dissolution medium is added and the experiment commences. The correlation between powder IDR (PIDR) data and DIDR data has been shown to be strong (r^2^ = 0.97) (11), suggesting that the less time-consuming powder measurements can be used to determine IDR accurately. In addition, this method can be used to determine the solubility (S) when excess material is used and a saturated solution is obtained. The ratio between PIDR and S is typically ~0.1, (11,12) as a consequence of the mathematical assumptions made when calculating the PIDR. A draw-back of the powder measurements is that particle size is not experimentally determined, but rather, has been estimated from the dissolution curve and the amount of drug used (11). When determining the surface area of the solid material, it is important to define the surface area involved in the dissolution process, i.e. the contact area between the solid material and the dissolution medium (13). The surface area of dry powder determined experimentally by various techniques is not necessarily equivalent to the area exposed during the dissolution. The surface area will depend on whether primary particles or agglomerates are characterized. As an example, for drugs that agglomerate extensively, the exposed surface area is much lower than the surface area of the primary particles. However, for particles that are well dispersed, the surface area of the primary particles has been shown to be satisfactory to use for interpretation of *in vitro* dissolution rate measurements (14).

Owing to the high number of poorly soluble compounds, the introduction of a variety of different technologies has been required to increase the solubility and dissolution, and to support absorption after oral administration. These technologies include the creation of solid dispersions, spray drying, the use of excipients, and particle size reduction. Although, there have been numerous activities during the early stage of development, a universal approach that covers these has not been adopted, usually because a large amount of compound is required for the technologies concerned. In a previous study, a “solubilization tool” was established tailored for *in vivo* studies. This technique uses wet-milling of compounds in aqueous medium to produce sub-micron suspensions. The suspensions are easily prepared and can be directly administered to animals (15,16). The controlled (sub-micron) suspensions can be used for both *in vitro* and *in vivo* studies, which will lead to less variability when bridging between the preformulation and preclinical studies. Another advantage is that controlled suspensions allow primary particles, and hence a larger surface area than powder and discs, to be in contact with the dissolution medium. Thus, the amount of compound dissolved per time unit is increased and dissolution measurements of poorly soluble compounds can be performed within shorter time frames.

In a previous work, we explored the extent to which DIDR is viable for poorly water-soluble compounds. The result showed the limitation of using disc dissolution when working with poorly soluble compounds (S_app_< 100 μg/mL) owing to the too low sensitivity of the μDISS Profiler, which relies on *in situ* UV readings (10). In addition, these measurements were time-consuming, typically requiring more than five hours to obtain a single DIDR value. In this case, dissolution assays from powder were recommended. Here, we investigated whether controlled suspensions produced by the solvent shift method or wet-milling would allow rapid and accurate dissolution profiling of poorly water-soluble compounds in the μDISS Profiler. To determine the accuracy of dissolution measurements from controlled suspensions, the IDR values from suspensions were compared to those from discs and powder for seven poorly water-soluble model drugs. A further aim was to standardize the dissolution rate measurements where variability due to differences in factors such as amount of compound added, aggregation of particles or disc compressing force were eliminated.

## Methods

### Materials

Aprepitant, cinnarizine, felodipine, fenofibrate, indomethacin and tadalafil were provided from different European Federation of Pharmaceutical Industries and Associations (EFPIA) partners within the Oral Biopharmaceutics Tools (OrBiTo) project. Griseofulvin, polyvinylpyrrolidone K30 (PVP K30), sodium dodecyl sulfate (SDS), (hydroxypropyl)methyl cellulose (HPMC) and dimethyl sulfoxide (DMSO) were purchased from Sigma-Aldrich (St. Louis, MO). FaSSGF/FaSSIF/FeSSIF powder was purchased from biorelevant.com (Croydon, UK).

### Preparation of Controlled Suspensions

Two different methods were explored with respect to the preparation of controlled suspensions: the solvent shift method making use of dilution of a highly concentrated DMSO stock solution and ball-milling of solid materials (17).

In the solvent shift method, controlled suspensions were prepared by precipitation of compounds when DMSO stock solutions were diluted in phosphate buffer with pH 6.5. The amount used to prepare the DMSO stock solution was calculated from the apparent solubility (S_app_) of the drug in fasted state simulated intestinal fluid (FaSSIF) and the decision to keep the DMSO concentration in the precipitation solvent low. For the compounds studied here, this resulted in 6.3–467.0 mg material dissolved in 50–500 μl DMSO. Between 10 to 250 μl of each stock solution was injected into the buffer, which also contained 0.2% (*w*/w) PVP K30. The final volume of the suspensions was 4–5 mL. The vials were placed in an ultrasonic bath during the addition of the stock solution whereupon the suspension was sonicated for 40 min.

In the ball-milling method, the suspensions were prepared by adding the compound and 10 milling beads (Ø 5 mm) to a milling bowl along with the phosphate buffer (pH 6.5) containing 1.0% (*w*/w) PVP K30 and 0.2% (*w*/w) SDS. When milling cinnarizine, 1.0% (*w*/w) HPMC was used instead of PVP K30, since milling with PVP K30 produced a semisolid material of cinnarizine. For indomethacin the buffer was adjusted to a pH of 2.5 to make sure that only a small fraction of the acidic compound was dissolved in the buffer. The suspensions were milled for 20 min at 600 rpm using a planetary ball mill (Model PM 100, Retsch, Germany). For compounds with a solubility value less than 10 μg/mL, a controlled suspension of 2 mg drug/mL buffer was made. For indomethacin, which had a solubility value greater than 300 μg/mL in FaSSIF, the concentration of the controlled suspension was 15 mg drug/mL buffer. For all other compounds, controlled suspensions of 4 mg drug/mL buffer were produced. All controlled suspensions were prepared on the day of the measurement.

### Characterization of Controlled Suspensions

The particle size of the suspensions was measured with a Zetasizer DS (Malvern Instruments, Worcestershire, UK). For the characterization, 50–100 μL of the controlled suspension was suspended in 1 mL of PhB (pH 6.5) and immediately inserted into the Zetasizer. The particle size was measured in triplicate and the mean value was used for the surface area calculations.

The solid form of the suspensions was evaluated using differential scanning calorimetry (DSC). Both the suspension produced by solvent shift and the wet-milled suspensions were filtered and dried in room temperature overnight (wet-milled material) or 37°C and 24 h (solvent shift precipitates) prior to the DSC measurement. Approximately 1 mg of the dried material was weighed into an alumina pan, and a ramp of 10°C per minute was used until a temperature 20°C greater than the literature Tm was reached (DSC Q2000, TA Instruments, Japan). The recorded thermograms were compared to the thermograms obtained using the same method on the received bulk (crystalline) drug.

The total surface area of all particles was estimated using the particle size and the density of the compound. General assumptions made were that the particle radius and number of particles were constant in the suspensions. The following equations were used:

2 $$ {\mathrm{V}}_{\mathrm{particle}}=\frac{4\uppi {\mathrm{r}}^3}{3} $$

Equation 2 was used to calculate the volume (V; cm^3^) of each particle, where r is the mean radius of the particles in the suspension with the assumption that the particles are spheres. The surface area (SA; cm^2^) was calculated using:

3 $$ {\mathrm{SA}}_{\mathrm{particle}}=4\uppi {\mathrm{r}}^2 $$

The volume (cm^3^) of the solid material used was determined from the total mass (m) added to the experiment and the density (ρ) of the compound.

4 $$ {\mathrm{V}}_{\mathrm{m}\mathrm{aterial}}=\frac{\mathrm{m}}{\uprho} $$

The total number of particles (n_particles_) added to each experiment was determined from the volume of the material and the volume of one particle.

5 $$ {\mathrm{n}}_{\mathrm{particle}\mathrm{s}}=\frac{{\mathrm{V}}_{\mathrm{material}}}{{\mathrm{V}}_{\mathrm{particle}}} $$

The total SA (cm^2^) of the solid material used in the experiment was obtained from Eq. 6, where the n_particles_ were multiplied with the surface area of each particle (SA_particle_).

6 $$ \mathrm{Total}\ \mathrm{SA}={{\mathrm{n}}_{\mathrm{particle}\mathrm{s}}}^{\ast }\ {\mathrm{SA}}_{\mathrm{particle}} $$

The total SA was calculated assuming monodisperse suspensions for all compounds except griseofulvin. Griseofulvin showed a bimodal particle size distribution, and here the total SA was calculated based on the average particle size of peak one and the average particle size of peak two, after adjustment for percentage of material found in these two fractions.

### Dissolution Studies from Discs, Powder and Controlled Suspensions

#### Establishment of Standard Curve

Dissolution testing in 15 mL FaSSIF original version (3 mM taurocholate and 0.75 mM lecithin) was performed for all compounds using discs, powder and controlled suspensions. Griseofulvin was used as a reference compound (18), and since its IDR was measured without taurocholate and lecithin in the reference literature, a phosphate buffer with pH 6.5 was used in this case. All experiments were performed at 37°C with a stirring of 100 rpm, in at least triplicate, using the μDISS Profiler (pION INC, MA). FaSSGF/FaSSIF/FeSSIF powder was used for the production of FaSSIF according to the protocol provided by the manufacturer (biorelevant.com, Croydon, UK). Standard curves were established to calibrate each probe used in the μDISS, for which aliquots of 5–10 μL of a DMSO-stock were added to 3 mL of FaSSIF. The interval of each standard curve, and hence the concentration of the DMSO-stocks, was dependent on the solubility of the compound in FaSSIF. After every aliquot addition (6–8 aliquots per standard curve), the solution was stirred for 1 min at 800 rpm before the concentration was determined.

#### Dissolution from Powder

Powder dissolution was performed according to a previously published protocol (12). Here, the material was weighed into vials and the measurement commenced at the same time as 15 mL preheated FaSSIF (37°C) was added to each vial. Each dissolution experiment was performed until the solubility plateau was obtained, typically resulting in measurements taking over three hours. All experiments were run at 37°C, stirred with a cross bar magnet (100 rpm) and performed in triplicate.

#### Dissolution from Discs

The Mini-IDR compression system (Heath Scientific, UK) was used to make miniaturized discs. A small amount of powder (about 5 mg) was loaded into the Mini-IDR and compressed for 2 min at 80 kg to obtain a disc with a surface area of 0.071 cm^2^. The discs were inserted into rotating disc carriers, placed into vials on a stirring heat block and the measurement was started at the same time as 15 mL of preheated FaSSIF (37°C) was added. Owing to the small surface area from the discs, between 1 and 7 h was required for each measurement. All experiments were run at 37°C using a stirring rate of 100 rpm and were performed in triplicate.

#### Dissolution from Controlled Suspensions

Dissolution measurements from controlled suspensions were made using different aliquots, again the volume used was dependent on the solubility of the compound in FaSSIF. For each compound, 2–3 different assays were run and each measurement was performed in triplicate, resulting in a total of 6–9 replicates for each compound. The prepared controlled suspensions were added to vials at an amount that was equal to 0.1–5.0 fold the saturation level after addition of the suspension to FaSSIF. Here, the FaSSIF was added to the vials, the run was started and a certain volume of suspension was added to each vial. For example, the concentration of the milled suspension was 4 mg/mL for felodipine. The S_app_ is approximately 30 μg/mL for felodipine in FaSSIF (10), and hence 450 μg can dissolve in the 15 mL FaSSIF. To obtain a final saturation level of 0.5, a volume of 56 μL (225 μg) was added to each vial. The dissolution testing with controlled suspensions was also performed using material to create saturated solutions. In the case of felodipine, 1 and 5 times the saturation level were used, where 112 μL (0.45 mg) and 560 μL (2.25 mg) were added in triplicate in two separate runs. The amount and concentration of FaSSIF was adjusted to the volume of the suspension added so that the final 15 mL volume corresponded to the original FaSSIF. For example, if 500 μL suspension was added (which was prepared in the corresponding blank buffer of FaSSIF, as described previously), we used 14.5 mL FaSSIF with a slightly higher concentration of taurocholate and lecithin to compensate for dilution effects. For this setup, the collection of data points was performed every other second for the first 5 min since the dissolution typically goes very fast at the beginning, and the calculation of suspension IDR (SIDR) will improve the more data points that are available. All experiments were run at 37°C and stirred with a cross bar magnet (100 rpm). The measurements were run for 20 min, but only the first minutes were used to calculate the SIDR.

### Calculation of the IDR

Data points obtained under the sink condition were used for the calculation of the IDR:

7 $$ \mathrm{IDR}={\mathrm{V}}^{\ast }{\mathrm{k}}^{\ast}\frac{1}{\mathrm{A}} $$

where k is the initial slope of the dC/dt curve (concentration in μg/mL per time unit) and A is the total surface area calculated (see section “Characterization of Controlled Suspensions”). The volume of FaSSIF was 15 mL in all experiments. A sliding k was used to estimate when the curve started to bend off (identified as a decreased k). As an example, the k of the first data points (e.g. data points 0–20) was calculated and compared to the k of data points 10–30, then data points 20–40 etc). When a decreased k was identified, all data points up until this part of the curve were used for the final calculation of k. The dissolution of griseofulvin and indomethacin was more or less instantaneous, so in this instance, only the first 3–5 data points were used for the IDR calculations as the dissolution curve started to bend off thereafter.

### Statistics

The solubility and IDR data are presented as the mean ± standard deviation. The SIDR is presented as 2–3 different measurements, using different amounts of compound. Every measurement was performed in triplicate, and hence, 6–9 IDR values are reported altogether for each compound. The statistical difference between the SIDR in FaSSIF for the different amount of compound added as well as the IDR measured using disc, powder and controlled suspensions were analysed with Anova using the general linear model, pairwise comparisons and the Tukey method.

## Results

### Particle Characterization

The solid material of the suspensions formed by solvent shift and wet-milling was investigated by DSC measurements (Table I). When the solvent shift method was used to prepare the suspensions, most of the compounds exhibited some solid state transformation (polymorph change or precipitating amorphous) as compared to the crystalline, starting material. The solid form of the compounds was not altered when exposed to milling. Therefore, the controlled suspensions prepared by milling were used in the dissolution experiments.

**Table I:** Melting Point of the Compounds Investigated; Original Material, Suspensions from Solvent Shift and Suspensions from Milled Material

Compound	Mw (Da)	logP	pKa	Tm Original (C°)	Tm solvent shift (C°)	Tm Milled(C°)
Aprepitant	534.4	4.5	9.7 (a); 2.8 (b)	252.9 ± 0.0	249.3 ± 0.5	252.6 ± 0.0
Felodipine	384.3	3.4	n	145.9 ± 0.1	139.0 ± 0.3	145.0 ± 0.2
Fenofibrate	360.8	5.3	n	81.1 ± 0.0	81.1 ± 0.1	81.3 ± 0.2
Cinnarizine	368.5	5.6	b (2.0; 7.5)	121.2 ± 0.1	112.9 ± 1.0	120.6 ± 0.2
Tadalafil	389.4	2.6	>10 (b)	301.6 ± 0.1	amorphous	301.8 ± 0.1
Indomethacin	357.8	3.1	4.1(a)	160.5 ± 0.2	142.7 ± 2.1	160.5 ± 0.1
Griseofulvin	352.8	2.2	n	219.6 ± 0.1	-	219.4 ± 0.1

**Fig. 1:**
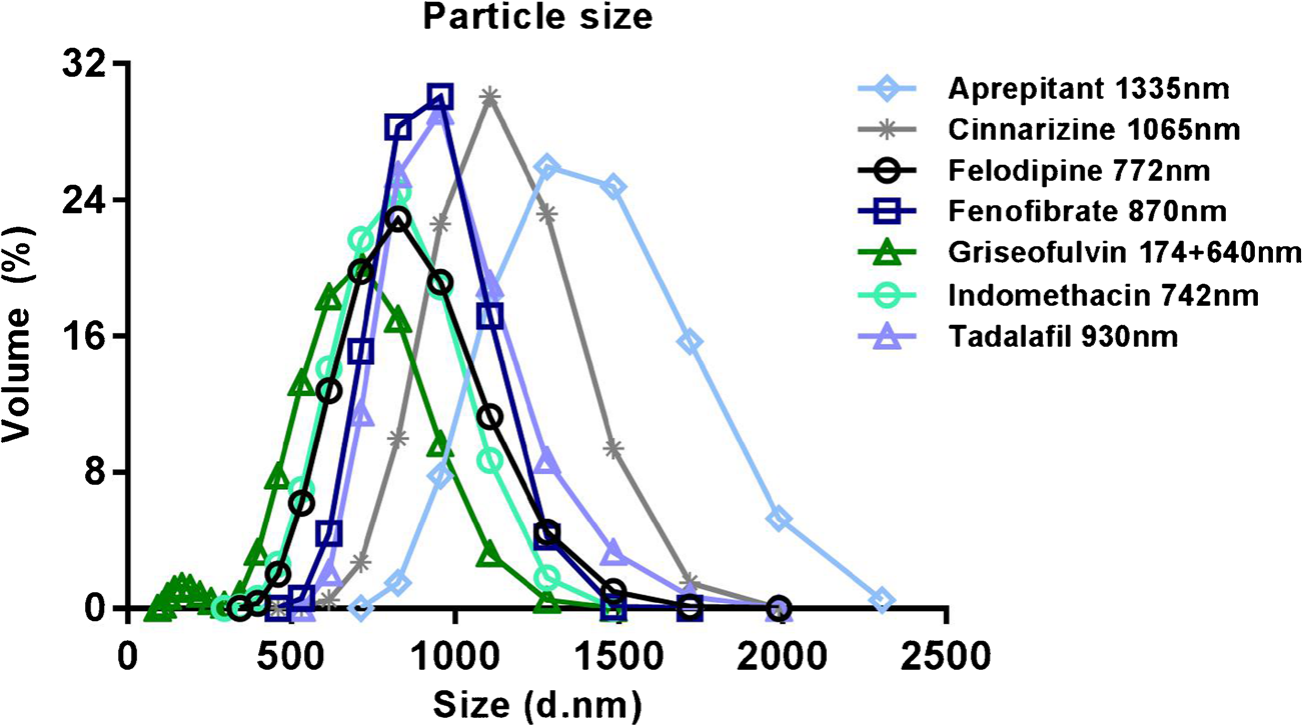
Particle distribution and mean particle size of all compounds for which IDR was determined from controlled suspensions.

The mean particle size of the controlled suspensions of all studied compounds is shown in Fig. 1. For most of the compounds the mean particle size was just below 1 μm.

### Dissolution Profiling

**Fig. 2:**
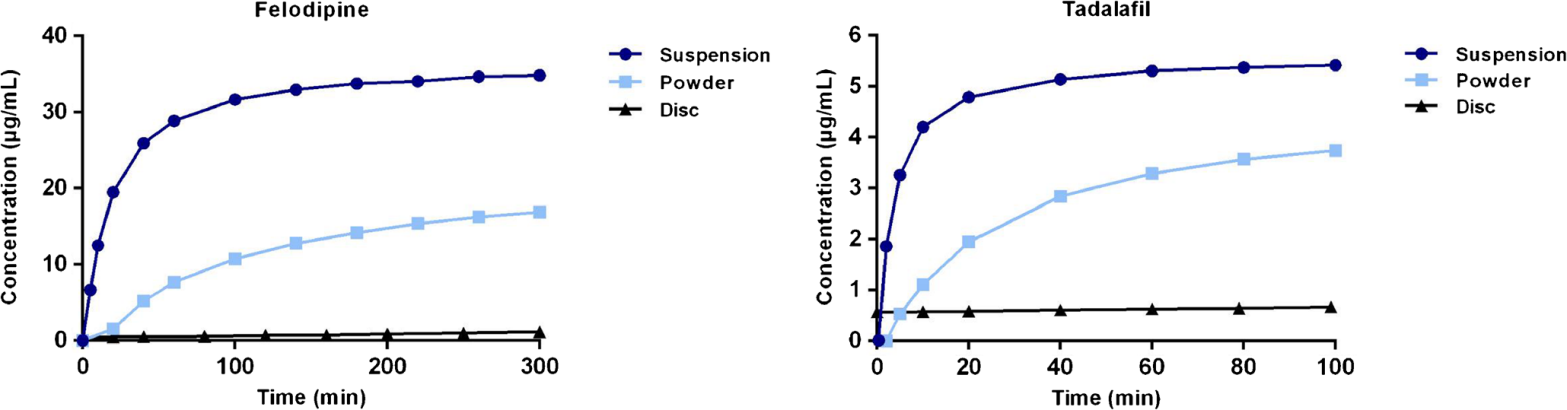
Dissolution profiles of felodipine and tadalafil in FaSSIF using suspensions (*dark blue circles*), powder (*light blue squares*) and discs (*black triangles*). The dissolution profiles from the disc measurements are slow because of the small surface area from the disc (0.071 cm^2^). The suspensions with milled particles have a more rapid dissolution. For clarity, the average data is presented for each ~20–30 min time point.

**Fig. 3:**
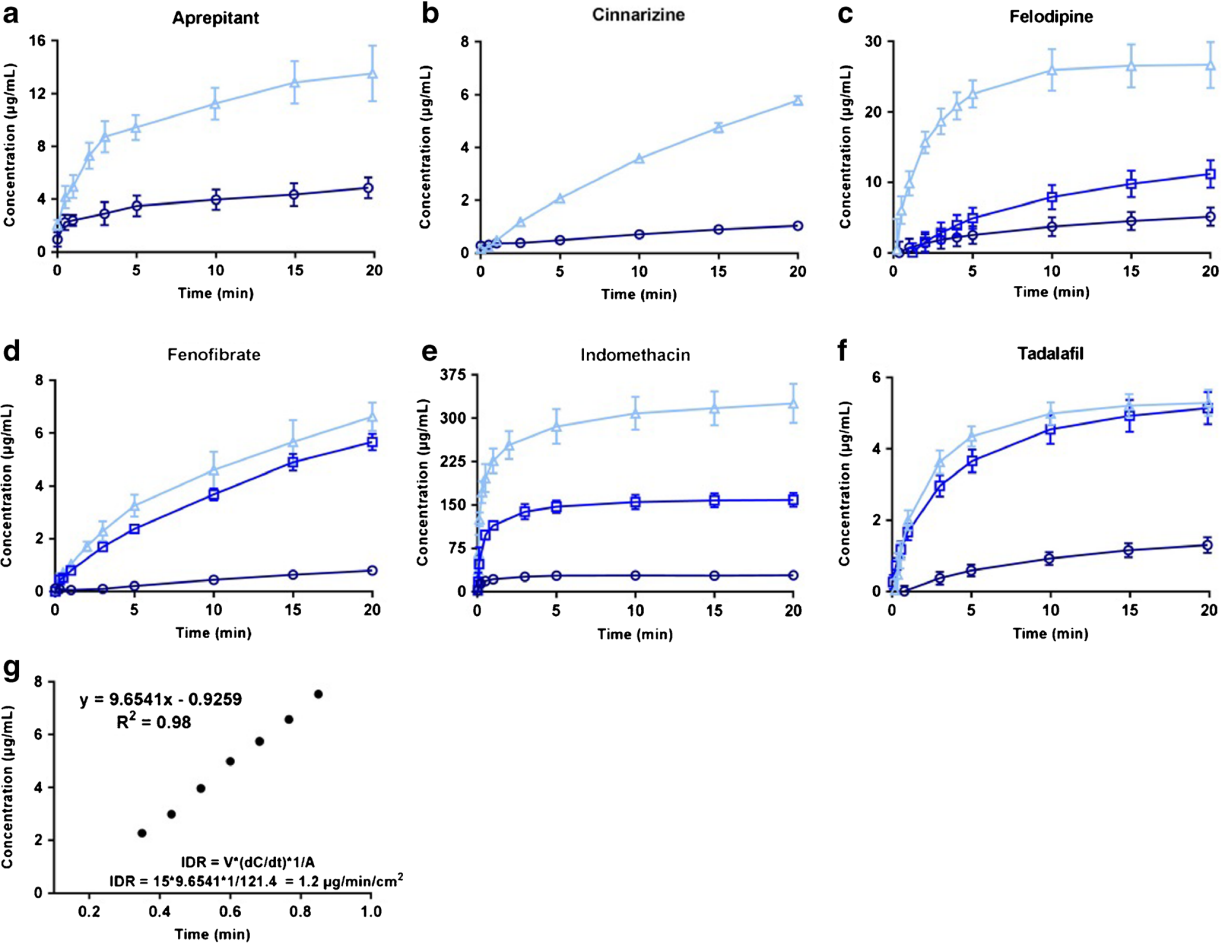
IDR measurements from controlled suspensions of (**a**) aprepitant (**b**) cinnarizine (**c**) felodipine (**d**) fenofibrate (**e**) indomethacin and (**f**) tadalafil using low (*circles*), medium (*squares*) or high (*triangles*) excess of each compound. Figure 3(**g**) illustrates the logic underpinning the IDR calculation from suspensions, here shown for felodipine (high excess of compound). The first time points are used to calculate the IDR based on the calculated surface area from the measured particle size.

The IDR and S_app_ of six model compounds in FaSSIF were measured using controlled suspensions, disc and powder (Table II). The dissolution profile, i.e. the time *vs*. concentration curve, was, as expected, dependent on the surface area of the compound exposed to the medium (shown for felodipine and tadalafil in Fig. 2). The time needed for dissolution profiling was as follows: disc based method > powder method > controlled suspension method. When comparing the IDR values obtained from controlled suspensions, discs or powder for each compound, the powder dissolution method was found to produce higher or lower IDR values than those obtained using discs (Table II). Here, the PIDR was extracted from the μDISS Profiler software and hence, the obtained value is dependent on the mathematical assumptions made therein. In contrast, the SIDR and DIDR values obtained from suspension and discs, respectively, corresponded well to one another. In addition, different saturation levels were studied using the controlled suspensions in which the material added resulted in either a non-saturated or a saturated solution upon complete dissolution (Fig. 3). The SIDR calculated from these different experiments were not statistically significantly different, and hence, the method can be used to accurately calculate IDR values regardless of the saturation level studied. If also the S_app_ is to be determined in the same experiments, excess material producing a saturated solution has to be used. All except tadalafil were statistically different (*p* < 0.01) when comparing PIDR to DIDR (Table II). In contrast, only tadalafil and felodipine were significantly different at this level (*p* < 0.01) when comparing SIDR and DIDR.

**Table II:** S_app_ and IDR in FaSSIF Measured Using Powder, Disc and Suspension-Based Methods

Compound	S_app_ (μg/mL)	PIDR (μg/min/cm^2^) Software	DIDR (μg/min/cm^2^) Calculated	SIDR (μg/min/cm^2^) Low saturation	SIDR (μg/min/cm^2^) Intermediate excess	SIDR (μg/min/cm^2^) High excess
Aprepitant	17.3 ± 1.8	1.3 ± 0.2	2.2 ± 0.7	1.0 ± 0.2	-	1.5 ± 0.2
Cinnarizine	11.1 ± 1.2	1.6 ± 0.2	0.1 ± 0.1	0.2 ± 0.0	-	0.2 ± 0.0
Felodipine	31.8 ± 1.7	4.4 ± 0.2	0.8 ± 0.2	1.0 ± 0.1	1.2 ± 0.1	1.2 ± 0.0
Fenofibrate	12.2 ± 0.3	1.22 ± 0.02	0.2 ± 0.0	0.3 ± 0.1	0.3 ± 0.0	0.2 ± 0.0
Griseofulvin^a^	10.7 ± 0.5	1.08 ± 0.05	10.1 ± 1.0	11.1 ± 1.3	10.1 ± 0.7	9.0 ± 1.1
Indomethacin	421.7 ± 17.6	42.8 ± 1.8	60.1 ± 5.6	70.6 ± 3.0	65.2 ± 4.8	63.2 ± 8.7
Tadalafil	5.9 ± 0.7	0.6 ± 0.1	0.5 ± 0.0	1.6 ± 0.3	1.8 ± 0.1	1.7 ± 0.1

The following abbreviations are used: Apparent solubility (S_app_), Powder IDR (PIDR), disc IDR (DIDR) and suspension IDR (SIDR)

^a^Griseofulvin was studied in buffer without any addition of taurocholate and lecithin

**Fig. 4:**
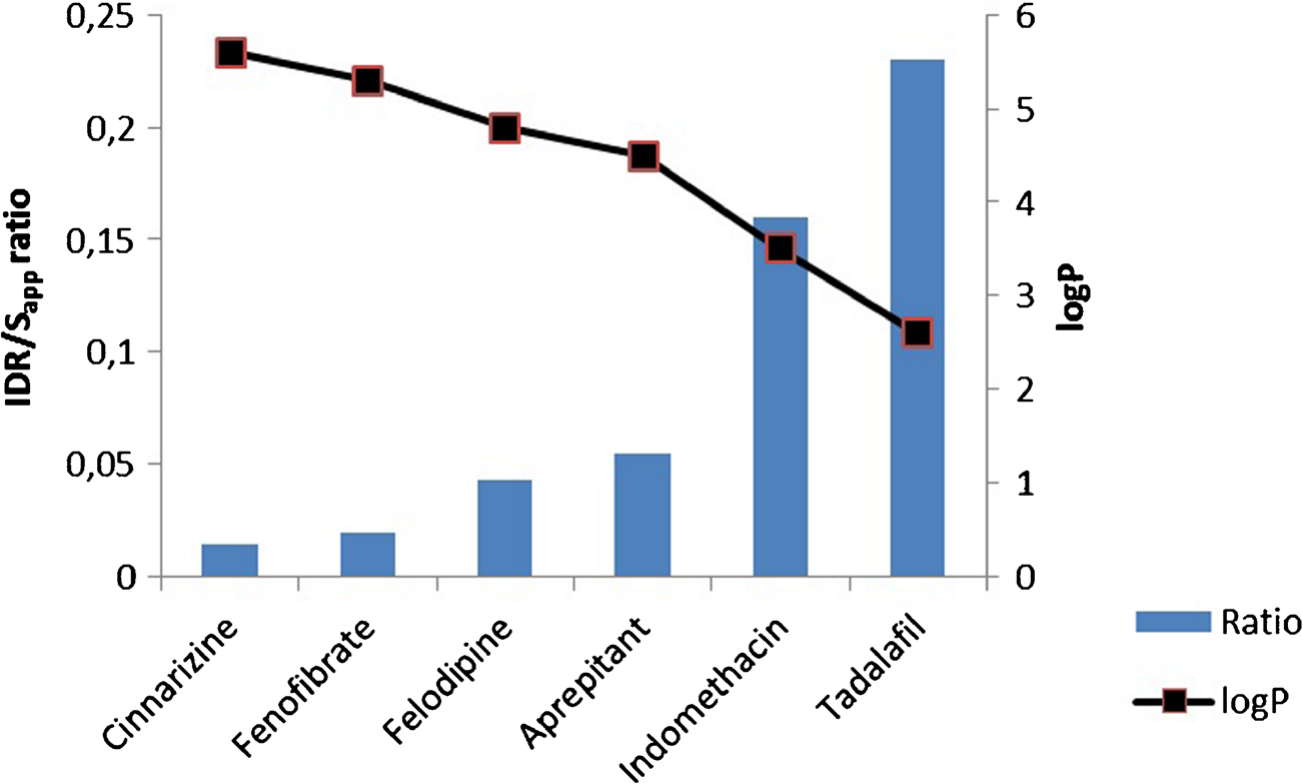
The IDR/S_app_ ratio compared to the logP values for the model compounds. A high ratio indicates a fast dissolution and a solubility limited compound. A low ratio indicates a slow dissolution and a dissolution rate limited compound.

The IDR/S_app_ ratio was calculated for the six compounds studied in FasSSIF (Fig. 4). The ratio showed an increasing trend with a decreasing logP value, i.e. the high logP compounds had the lowest IDR/S_app_ ratio, indicating that the dissolution rate was the major limiting factor for these compounds. The particle size needed for the compounds to be completely dissolved in the intestine, using a transit time of 3.32 h (19), was calculated and three of the six compounds were found to demand particle sizes below 10 μm to address the dissolution-limited absorption (Table III).

**Table III:** The Particle Size Needed for the Model Compounds to Dissolve Completely in 3.32 h, Based on the Maximum Dose Administered for Each Compound

Compound	Dose (mg)	1 μm	10 μm	25 μm	50 μm	100 μm	Size (μm)
Cinnarizine	25	Yes	No	No	No	No	2.0
Fenofibrate	200	Yes	No	No	No	No	3.1
Aprepitant	125	Yes	No	No	No	No	7.2
Felodipine	10	Yes	Yes	No	No	No	11.3
Tadalafil	20	Yes	Yes	No	No	No	13.5
Indomethacin	100	Yes	Yes	Yes	Yes	Yes	600.0

The maximum doses were obtained from the Swedish Physician Desk Reference. Material being completely dissolved=yes; not dissolved=no. Size provided in right column shows which particle size is needed to allow all material to dissolve within 3.32 h

## Discussion

Dissolution tests that better predict *in vivo* performance of a drug would be of great value to shorten time needed for formulation development as well as reduce the number of clinical studies required. With this in mind, more rapid and accurate dissolution measurements may be obtained by making use of controlled suspensions (17). In this study we show time saving effects by using suspensions for IDR measurements of poorly soluble compounds; only minutes are needed to obtain an accurate IDR comparable to the DIDR. As a consequence of the low concentration and of limited detection when measuring the IDR of poorly soluble compounds using discs, such studies can take several hours, if they are measurable at all with *in situ* UV readouts. Other difficulties associated with the disc method are that discs can be difficult to compress owing to stickiness of the compound, uneven disc surfaces may be obtained during compression, and erosion of the compressed disc may occur during the dissolution measurement. Another reason for using suspensions instead of powder or discs is that the first *in vivo* studies usually are performed using suspensions, administered with different dose strengths. Controlled suspensions may therefore be a good way to bridge between *in vitro *dissolution and *in vivo *absorption studies. Ideally this *in vitro* method may be used to tailor the absorption and hence the plasma concentration attained. Finally, agglomeration is reduced for suspensions compared to powder dissolution studies, and therefore the exposed surface area during the dissolution study is better defined.

The controlled suspensions make it easy to standardize measurements since the same suspension can be used for several measurements, e.g. for studies of fasted and fed conditions or prototype formulations, adding the same volume and amount of compound in each run. If dissolution profiles are to be compared, it is important to take the added material into consideration. The same amount of material should always be added to every measurement when the purpose is to compare the dissolution rate. Here, a controlled suspension is a good alternative where the same volume is pipetted to every vial with, e.g., a biorelevant medium, making it fast and easy to perform with high reproducibility. For this purpose it is also advantageous to use a technique that may determine concentrations at short time intervals to obtain good measurement of the initial dissolution. For this purpose the μDISS Profiler is a useful technique with its *in situ* UV-probes that enable frequent collection of data points (every second).

In this study, the IDR from controlled suspensions is measured, making use of the initial slope. Only the first few minutes of the slope are used to make sure that the sink condition is maintained, with the assumption that the surface area is constant during this time. In this work, the IDR was calculated based on a sliding slope. However, in the case of indomethacin and griseofulvin, with rapid dissolution, it was only possible to use the first 3–5 time points for the calculation; indeed it has previously been reported that indomethacin is suitable to study with discs owing to its rapid dissolution (10). As a rule of thumb the SIDR method is a good method to use for compounds with solubility <100 μg/mL, in accordance with the recommendations of when powder dissolution studies should be used rather than disc studies, when using the μDISS (10). The IDR values from controlled suspensions and discs were compared and they show good agreement (Table II). This further strengthens the result that the sample-efficient and time-saving IDR measurements from controlled suspensions are a good alternative to the disc measurements. As an example, the SIDR will decrease the amount used from 5–10 mg to amounts as low as 75 μg and decrease the time needed for measurements from hours to only a couple of minutes.

In our study we used a wet-milling technique to produce controlled suspensions with a mean particle size of approximately 1 μm. Further, the surface area was calculated from the density and the amount added to the measurements, with the assumption that the particles are spherical. However, other approaches can be used, for example if the compound is sensitive to milling or if larger particles sizes need to be used. Here, the important factor to control is the actual surface area of the material added when the measurement was initiated and to make sure that the particles do not agglomerate in solution. In addition, the technique used should not induce solid state transformation, which we observed the solvent shift technique did for many of the model compounds. The straightforward, simplified calculations of the surface area based on the particle size and density performed surprisingly well. Felodipine and fenofibrate are illustrative examples of when the SIDR values calculated are between 1.0–1.2 μg/min/cm^2^ and 0.2–0.3 μg/min/cm^2^, respectively, for the different amounts of the compounds added. This can be compared to the DIDR of felodipine and fenofibrate which is 0.8 μg/min/cm^2^ and 0.2 μg/min/cm^2^, respectively (Table II). When wet-milling a compound, the particles are reduced at the same time as the polymer/surfactant-solution acts to disperse the particles and hence, the particles of the suspension do not agglomerate. A similar wet-milling approach has been shown to reduce the size of the particles to approximately 1 μm, if the milling is performed for longer than 10 min (16). To further reduce the size of the particles (below 1 μm), a longer milling time or a higher speed needs to be applied. However, milling may produce the amorphous form via mechanical activation, but typically this requires significantly longer time scales (20). In this study, the thermograms showed no changes in the thermal properties of the milled material compared to those for the received materials, which indicates that the compounds are still in their original crystalline form after this processing.

To validate our method, every compound was measured 2–3 times (in triplicates) using different volumes of suspension (Fig. 3). Here, the aim was to make sure that, regardless of the amount of compound used in the measurement, the same IDR value would be obtained. The measurements were performed in FaSSIF, and since FaSSIF already contains ingredients working as surfactants, the small amounts of PVP and SDS added to the experiment were assumed to have negligible effect on the dissolution. This was confirmed through powder and disc measurements in FaSSIF, adding the same amount of PVP and SDS as in the suspension measurements (data not shown). If other conditions are used for SIDR measurements, i.e. other dissolution media such as simulated gastric fluid or plain buffers, similar evaluation needs to be performed to reveal potential effects of low concentration of polymer and surfactant on the resulting IDR and S_app_.

Griseofulvin was selected as a reference compound based on an earlier study performed by Mosharraf and Nyström (18). In their study, the dissolution rate was investigated using a known surface area. Their study was performed in 0.9% *w*/w NaCl with 0.01% *w*/w Tween 80, hence we only studied griseofulvin in buffer without the taurocholate and lecithin. The pH of their medium was not stated, but since griseofulvin is a neutral compound, pH should not have an effect on the solubility or the dissolution rate. Our SIDR for griseofulvin was compared to the value Mosharraf and Nyström obtained; these were 9.0–11.1 μg/min/cm^2^ and 11.2–13.7 μg/min/cm^2^, respectively. Hence, the data from griseofulvin confirmed that the SIDR method developed here produce data that are in agreement with other SIDR methods available.

The resulting data for the IDR and S_app_ can be used to provide information on whether it is likely that absorption will be dissolution rate limited. Figure 4 shows the IDR/S_app_ ratio of the six compounds studied in FaSSIF, where the lowest ratio is 0.015 (cinnarizine) and the highest 0.23 (tadalafil). As a guideline, when the compound shows dissolution rate-limited absorption, we expect the ratio to be low and when the compound mainly is limited by the solubility the ratio to be high. However, this needs to be set in the context of the dose and the particle size. Since the dose is not known in the early state of drug development, we introduce the IDR/S_app_ ratio as a tool to reveal whether a compound may be expected to be dissolution rate-limited or not, and to determine to what extent particle size reduction would drive the absorption.

The Developability Classification System (DCS) has been introduced to distinguish between solubility-limited and dissolution rate-limited drugs (19). To compare the reference compounds, the tendency to be solubility and/or dissolution rate limited was evaluated using a volume of 250 mL and the dose for each compound. Table III shows the actual size the compounds need to have to dissolve completely during transfer in the intestine. For these calculations a transit time of 3.32 h was used and sink condition was assumed to apply. The six model compounds were further explored for potential dissolution rate limited absorption based on their respective dose, particle sizes between 1 and 100 μm and an estimated transit time of 3.32 h. This was done to make the particle size reduction effects on absorption visible. For example, cinnarizine, with the lowest IDR/S_app_ ratio, has an extremely poor dissolution and is dissolution rate-limited when a particle size >2 μm is used, based on a dose of 25 mg. Owing to the poor solubility of cinnarizine, the compound is also solubility-limited. The ranking of the model compounds in Fig. 4 (IDR/S_app_ ratio) and Table III (the particle size needed for complete dissolution) correspond well with each other. However, aprepritant and felodipine have switched place in Table III as a consequence of the large difference in dose for these two compounds. Indomethacin also dissolves faster, because of its relatively high solubility (421 μg/mL) compared to the other model compounds. This shows that the solubility needs to be taken into consideration too, when analyzing the data based on the IDR and the IDR/S_app_ ratio.

Another trend that can be seen in Fig. 4 is that of the logP values of the compounds. The compounds show a decreasing logP value with an increased IDR/S_app_ ratio. In biorelevant media the relation between IDR and S_app_ will also be a result of the solubilization in the mixed micelles present. There is a strong relationship between the solubilization and the drug lipophilicity; the higher the logP the higher solubilization to expect (12). However, the dissolution rate does not increase to the same extent due to the slower diffusion of micelles than the monomer drug, and hence, the increase in solubility as a result of solubilization will drive a lower IDR/S_app_ ratio. Another complicating factor is that of biorelevant buffer capacity. If a medium with biorelevant buffer capacity is used, the drug may buffer the medium during the dissolution and cause a pH shift within the unstirred water layer surrounding the particle surface (21). This would then also result in a lower IDR than expected from the solubility, and a lower IDR/S_app_ ratio. Therefore, when analyzing factors limiting absorption of BCS class II compounds, all these factors (IDR, S_app_, IDR/S_app_ and dose) merit attention and should be evaluated in concert; preferably making use of BDMs with biorelevant buffer capacity and with bile components present when studying lipophilic, ionizable poorly water-soluble drugs.

Several useful measures were introduced by Amidon *et al*. when the BCS was established (1) and later by Butler and Dressman when the DCS was introduced (19). Of particular interest for this work are the dose number, dissolution number and time for dissolution. Below we visualize how the IDR can be used to calculate similar properties. If the IDR and the surface area are known, dC/dt (μg/mL/min), i.e. the slope of the dissolution curve under sink condition (k), can be calculated using Eq. 8.

8 $$ \mathrm{k}=\frac{{\mathrm{IDR}}^{\ast}\mathrm{A}}{\mathrm{V}} $$

The IDR can be used to calculate the time needed for a dose to be completely dissolved (t_diss_) using Eq. 9. The time calculated is a “best case” scenario, assuming the drug to be rapidly absorbed.

9 $$ {\mathrm{t}}_{\mathrm{diss}}=\frac{\frac{\mathrm{Dose}}{\mathrm{V}}}{\mathrm{k}} $$

**Fig. 5:**
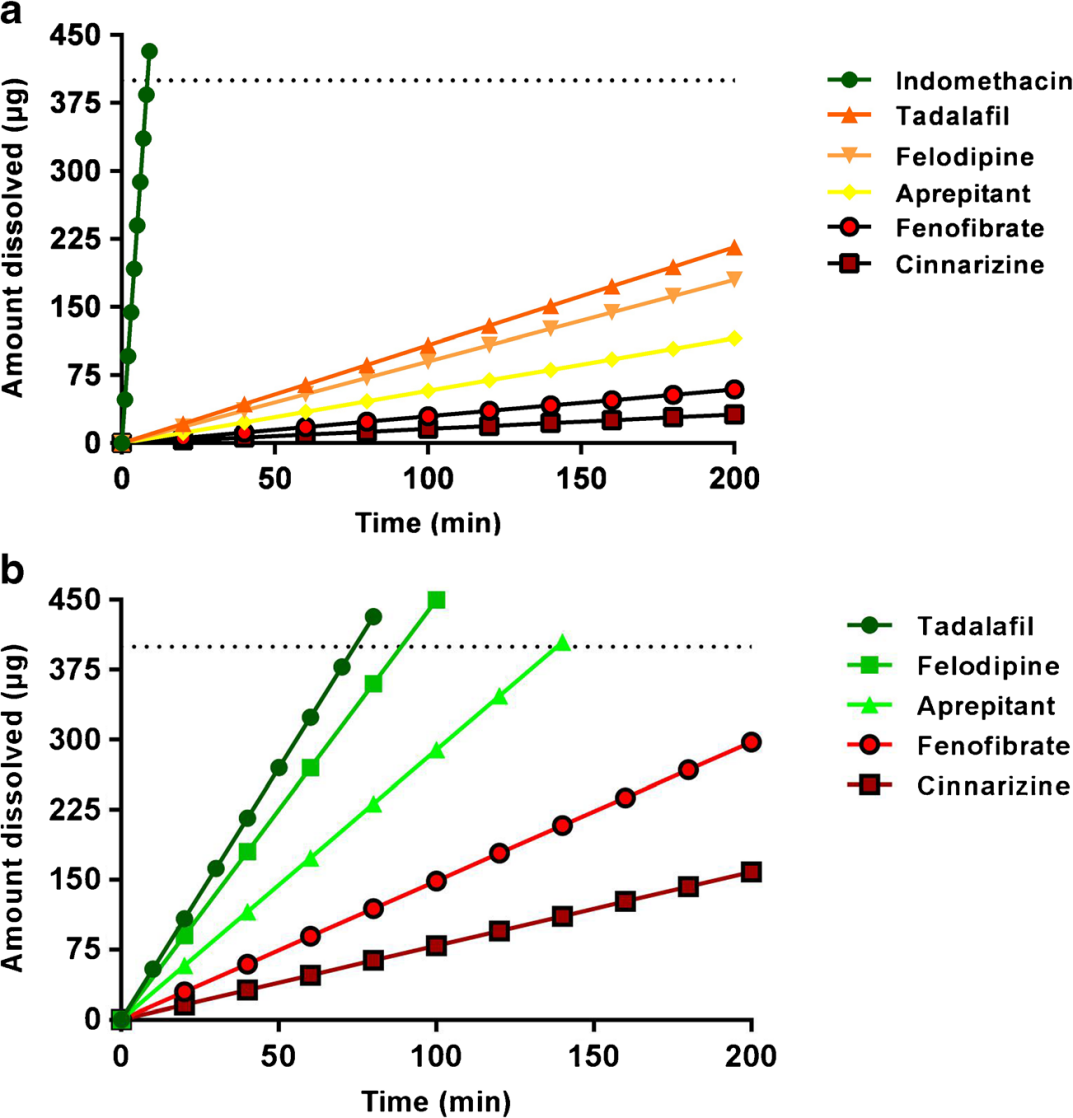
Dissolution of the six model compounds studied in FaSSIF using (**a**) a dose of 100 mg and a particle size of 25 μm and (**b**) a dose of 100 mg and a particle size of 5 μm. The time needed for complete dissolution has been calculated using the IDR, a transit time of 3.32 h (~200 min) (19) and a volume of 250 mL. The compounds with values above the dotted line at 400 μg are completely dissolved during the transit time.

As an example of how this strategy can be used during preformulation, we made use of the data obtained from the six model compounds and ‘generic’ doses and particle sizes. Figure 5a shows the dissolution profile of the reference compounds using 100 mg as the standard dose, a particle size of 25 μm as the default particle size and 250 mL as dissolution volume. Here, the only compound being completely dissolved within the given transit time of the small intestine is indomethacin (8.3 min).

If, instead, a particle size of 5 μm is used, tadalafil (74 min), felodipine (90 min) and aprepritant (138 min) also dissolve completely during the transit of the small intestine. Cinnarizine and fenofibrate need further milling (Fig. 5b). This is also visible in Fig. 4 where cinnarizine and fenofibrate are the two compounds with the lowest IDR/S_app_ ratio. However, these calculations rely on that the compound is primarily dissolving in the small intestine. This is true for neutral and acidic compounds, whereas weak bases typically dissolve in the gastric compartment. For the compounds studied herein, the gastric compartment has been shown to be the most important compartment for dissolution (and source of interindividual variability in bioavilability) for cinnarizine (22). This point towards the need to set the calculations based on the IDR and S_app_ in the perspective of the pH-dependent solubility.

## Conclusion

We conclude that faster and more accurate dissolution profiling can be performed when using controlled suspensions with dispersed primary particles instead of the standard powder dissolution experiment performed with the μDISS. The controlled suspensions allow a larger, well-defined surface area to be in contact with the dissolution media than that obtained for discs and powder. This will increase the amount dissolved per time unit and hence facilitate IDR measurements of poorly soluble compounds in shorter time frames. For the six model compounds studied in FaSSIF, the IDR/S_app_ ratio varied from 0.015–0.23. We suggest that this ratio is a valid and convenient measure for identifying if a compound is likely to show dissolution rate-limited absorption and hence is sensitive to particle size reduction. By making use of the IDR, the time needed for a compound to completely dissolve (under sink conditions) can be calculated from a presumed dose and particle size. Likewise, the particle size needed to dissolve a dose completely can be calculated using the IDR. The features of the method make it a proper bridge between *in vitro* and *in vivo* measurements during the early development of poorly water-soluble compounds.

## Abbreviations

BCS: Biopharmaceutics classification system
DCS: Developability Classification System
DIDR: Disc intrinsic dissolution rate
DMSO: Dimethyl sulfoxide
DSC: Differential scanning calorimetry
EFPIA: European federation of pharmaceutical industries and associations
FaSSIF: Fasted state simulated intestinal fluid
HPMC: (hydroxypropyl)methyl cellulose
IDR: Intrinsic dissolution rate
OrBiTo: Oral biopharmaceutics tools
PIDR: Powder intrinsic dissolution rate
PVP K30: Polyvinylpyrrolidone K30
SA: Surface area
S_app_: Apparent solubility
SDS: Sodium dodecyl sulfate
SIDR: Suspension intrinsic dissolution rate
